# The Depression Network (DeNT) Study: methodology and sociodemographic characteristics of the first 470 affected sibling pairs from a large multi-site linkage genetic study

**DOI:** 10.1186/1471-244X-4-42

**Published:** 2004-12-09

**Authors:** Anne Farmer, Gerome Breen, Shyama Brewster, Nick Craddock, Mike Gill, Ania Korszun, Wolfgang Maier, Lefkos Middleton, Ole Mors, Mike Owen, Julia Perry, Martin Preisig, Marcella Rietschel, Theodore Reich, Lisa Jones, Ian Jones, Peter McGuffin

**Affiliations:** 1 Medical Research Council Social Genetic and Developmental Psychiatry Centre, Institute of Psychiatry, London, UK; 2 Glaxo Wellcome Research and Development, Greenford UK; 3 Dept of Psychological Medicine, University of Wales, College of Medicine, Cardiff UK; 4 Department of Psychiatry, Trinity Centre for Health Sciences, Dublin Eire; 5 Barts and The London, Queen Mary's School of Medicine and Dentistry, London UK; 6 Department of Psychiatry, University of Bonn, Bonn, Germany; 7 Department of Psychiatry, University of Aarhus, Aarhus, Denmark; 8 University of Lausanne, Faculty of Biology and Medicine, Hopital de Cery, Unite psychopathologie, Prilly, Switzerland; 9 Zentralinstitut for mental health, Mannheim, Germany; 10 Dept of Psychiatry, Washington University, St Louis, USA

## Abstract

**Background:**

The Depression Network Study (DeNt) is a multicentre study designed to identify genes and/or loci linked to and/or associated with susceptibility to unipolar depression in Caucasian families. This study presents the method and socio-demographic details of the first 470 affected sibling pairs recruited from 8 different sites in Europe and the United States of America.

**Methods:**

Probands fulfilling either the Diagnostic and Statistical Manual 4^th ^edition (DSM-IV) or the International Classification of Diseases 10^th ^edition (ICD-10) criteria for recurrent unipolar depression of moderate or severe degree and who had at least one similarly affected sibling were eligible for the study. Detailed clinical and psychological assessments were undertaken on all subjects including an interview using the Schedules for Clinical Assessment in Neuropsychiatry. Blood samples were collected from all participants to extract DNA for linkage analysis.

**Results:**

The different sites used different recruitment strategies depending on local health care organisation but despite this there was remarkable similarity across sites for the subjects recruited. Although the Bonn site had significantly older subjects both for age of onset and age at interview, for the sample as a whole, subjects were interviewed in their mid-40s and had experienced the onset of their recurrent depression in their 20s. Preliminary genome screening was able to include 929 out of the 944 subjects (98.4%) typed at 932 autosomal and 544 X chromosome markers

**Conclusions:**

This paper describes the methodology and the characteristics of the subjects from the 414 families included in the first wave of genotyping from the multi-site DeNT study. Ultimately the study aims to collect affected sibling pairs from approximately 1200 families.

## Background

Genetic risk factors are well established for major affective disorders and a recent twin study has suggested that unipolar depression has a stronger genetic influence than was previously thought. McGuffin and colleagues [1] have estimated that the heritability (i.e. the proportion of liability explained by genetic risk factors) may be over 70% in a clinically ascertained twin sample while a population based twin study resulted in a very similar estimate using a re-test method of assessing lifetime diagnosis [2].

The majority of studies suggest a relative risk to siblings (λs) of affective disorder is in the region of 3 [3]. However, a recent study comparing the siblings of unipolar depressives with the siblings of healthy controls using strict definitions of both depression and health found a substantially higher λs of over 9 [4].

The inheritance of unipolar depression is complex and involves an inter-play of genetic and environmental factors. For unipolar depression these include certain types of severe and threatening life events such as events associated with humiliation or loss [5,6].

Despite an excess of females to males of about 2 to 1 for unipolar depression, the heritability in a clinically ascertained sample was the same in men and women [1]. Some population based twin studies suggest at least some of the genes conferring liability differ between the sexes [7] while others do not [8]. Although it has been suggested that early onset depression is more clearly familial than later onset, this is not supported by a meta-analysis [9]. The only characteristics of probands associated consistently with higher familiality or heritability are recurrence of episodes and severity of disorder [1,9].

### Previous linkage studies of unipolar depression

Most previous linkage studies have been carried out in families identified by a bipolar proband and where unipolar and bipolar relatives are frequently grouped together into a broad definition of affective disorders. Most such studies have focussed on multiple affected extended pedigrees on the assumption that there may be a sub-set segregating a gene of major effect. This approach has been successful in complex disorders such as early onset Alzheimer's disease and breast cancer. However, consistent evidence of major gene effects in bipolar disorder has not been forth-coming[10]. In addition, the unknown mode of inheritance creates inherent difficulties in classic linkage approaches and consequently sib-pair methods are attractive in the study of complex familial disorder. An affected sib pair genome scan study of recurrent depression has now been published suggesting that there is a depression susceptibility locus on chromosome 15q [11]. Another genome scan focusing on multiply affected families found the strongest evidence for linkage on chromosome 12q [12]. In addition a genome scan of multiply affected families with alcoholism and in whom some individuals had depression or co-morbid alcoholism and depression found evidence of a depression linked locus on chromosome 1p. Clearly these results require further scrutiny and replication.

## Methods

### Subjects

Sibling pairs affected with recurrent unipolar depression were recruited from 8 clinical sites: Aarhus, Denmark; Bonn, Germany; Dublin, Ireland; Lausanne, Switzerland; St Louis, USA and London, Cardiff and Birmingham, UK. In addition, where available, parents of the affected sibling pairs were also included in the study.

Subjects were identified from psychiatric clinics, hospitals, general medical practices and from volunteers responding to media advertisements. Caucasian subjects over the age of 18 were included if they had experienced 2 or more episodes of unipolar depression of at least moderate severity separated by at least 2 months of remission as defined by the Diagnostic and Statistical Manual 4^th ^edition operational criteria (DSMIV) [13] or the International Classification of Diseases 10^th ^edition operational criteria (ICD10), for unipolar depression [14]. Probands were all white and of white European parentage. They were included in the study if they had at least one biological sibling, not a monozygotic twin, over the age of 18 years meeting the same diagnostic criteria. Subjects were excluded if either sibling had ever fulfilled criteria for mania, hypomania or schizophrenia.

Subjects were also excluded if they experienced psychotic symptoms that were mood incongruent or present when there was no evidence of a mood disturbance. Other exclusion criteria were intravenous drug use with a lifetime diagnosis of dependency; depression occurring solely in relation to alcohol or substance abuse or depression only secondary to medical illness or medication, and a clear diagnosis of bipolar disorder, schizophrenia, schizo-affective disorder or acute or transient psychotic disorders in first or second-degree relatives.

### Clinical assessment

All subjects were interviewed using the Schedules for Clinical Assessment in Neuropsychiatry (SCAN) [15,16]. Items of psychopathology in the SCAN interview were rated for presence and severity according to the worst and second worst episodes of depression identified by the subjects. For the purposes of rating severity, subjects were asked to identify within each of these episodes of depression a 4–6 week period when their symptoms were at their worst (peak intensity). The majority of the SCAN items were coded as follows; 0 – indicates absence of the item, 1 – the item was present but to a mild degree or intermittently throughout the peak intensity 4–6 weeks, 2 – item moderately severe and present for more the 50% of the peak intensity period or severe but present for less than 50% of the peak intensity period, 3 – item severe for more than 50% of the peak intensity period. The computerised version of the SCAN2.1 is built on top of the IShell system, which is a computer aided personal interviewing tool produced by the World Health Organisation [17] and which provides diagnoses according to DSMIV and ICD10 operational definitions.

### Interviewer training and reliability across sites

All interviewers from each site attended a 4-day SCAN training course in the UK. Each site also undertook further inter-rater reliability meetings regularly and annually all interviewers from all sites took part in a joint inter-rater reliability exercise.

### Ethical approval

All sites obtained ethical approval for the DENT study within their own countries and institutions. All study participants gave written informed consent for participation in the study.

### Self-report questionnaires and other information collected from participants at interview

In addition to the SCAN interview all study participants completed the Eysenck Personality Questionnaire [18] and a detailed family history of psychiatric and physical disorders. For the 6 months prior to the worst episode and 6 months prior to the second worst episode as well as the 6 months prior to interview the Brief Life Events Questionnaire (BLEQ) identified which of 12 types of severe life events had occurred. These were based on the list proposed by Brugha and colleagues [19] to which childbirth was added. If such an event had occurred the subject was also asked to rate the impact of the event as; very distressing (a score of 3), moderately distressing (scored 2) or not very distressing (scored 1). The BLEQs therefore gave a summated impact score out of 39 for each 6-month time frame.

### Blood samples

At the time of the SCAN interview interviewers obtained 25 ml of whole blood that was collected in 37.5 ml (EDTA containing) monovettes. In addition drops of blood were placed on a Guthrie blood spot card. The blood samples were labelled with a bar code, gently mixed and stored frozen upright in a -20 degree centigrade freezer pending DNA extraction.

### Phenotypic data analysis

All phenotypic information from interviews and questionnaires was coded by assigning a number to each subject, and removing any personal identifying information. The same codes were used on the blood sample tubes using a bar code system. The phenotypic information was first entered on an EXCEL spread sheet after which a data file was created using Statistical Procedures for the Social Sciences (SPSS) version 10 for Windows for the statistical analyses.

## Results

### Inter-rater agreement for SCAN interview

All the interviewers from all sites took part in a joint inter-rater reliability exercise (in English) involving both audio-taped interviews with study subjects and videotaped interviews with actors, role-playing a depressed subject. Item by item kappa statistics for SCAN items, were calculated comparing each interviewer's ratings against AF's "master" rating. A mean item by item kappa coefficient across all the sites of 0.77 (range 0.63 – 0.89) was obtained indicating a substantial level of inter-rater agreement.

### Number of subjects, age at interview, age at illness onset and gender by site

For inclusion in the first part of the linkage analysis, 944 affected subjects were genotyped from the 8 study sites as follows: Aarhus 48, Birmingham 146, Bonn 110, Cardiff 126, Dublin 154, Lausanne, 56, London 111 and St Louis 193. The age at interview and age of illness onset by gender of the subjects recruited at each site are shown in Table 1.

**Table 1:** Numbers of male and female subjects, age at interview and age of illness onset by site

**Site**	**Gender**	**Number**	**Age at interview (SEM)**	**Age at illness onset (SEM)**
Aarhus	Female	30	43.53 (2.1)	21.39 (1.8)
	Male	18	45.11 (2.0)	24.67 (1.9)
Birmingham	Female	104	48.12 (1.3)	23.44 (1.1)
	Male	42	49.24 (2.2)	26.19 (1.7)
Bonn	Female	86	51.67 (1.3)	28.10 (1.9)
	Male	24	50.67 (2.9)	24.24 (4.1)
Cardiff	Female	85	43.55 (1.1)	23.76 (1.3)
	Male	41	45.12(1.9)	22.92 (1.6)
Dublin	Female	110	42.86 (1.2)	21.12 (1.0)
	Male	44	44.43 (0.9)	24.97 (2.1)
Lausanne	Female	43	48.67 (1.5)	24.69 (2.0)
	Male	13	42.39 (2.0)	25.3 (2.1)
London	Female	80	45.04 (1.1)	22.63 (1.4)
	Male	31	47.29 (2.1)	20.71 (2.0)
St Louis	Female	132	47.14 (1.0)	18.44 (0.9)
	Male	61	47.15 (1.6)	17.58 (1.5)

SEM = Standard Error of the Mean

Mean age at interview for both sexes combined for each site were as follows: Aarhus 44.13 years (standard error of the mean (SEM)1.5), Birmingham 48.44 years (SEM 1.1), Bonn 51.46 years (SEM 1.2), Cardiff 44.06 years (SEM 0.9), Dublin 43.32 years (SEM 1.0), Lausanne 47.21 years (SEM 1.3), London 45.67 years (SEM 1.0), St Louis 47.14 years (SEM 0.9). These mean age differences were statistically significant (Analysis of variance(ANOVA): F = 6.26 degrees of freedom (df) 7, 936, p < 0.001. Tukey Post hoc test: Dublin, Cardiff, Aarhus, London, St Louis, Lausanne, Birmingham < St Louis, Lausanne, Birmingham, Bonn).

Mean age at illness onset for both sexes combined per site were as follows: Aarhus 22.67 years (SEM 1.3), Birmingham 24.30 years (SEM 0.8), Bonn 27.28 years (SEM 1.2), Cardiff 23.47 years (SEM 0.9), Dublin 22.27 years (SEM 0.8), Lausanne 24.85 years (SEM 1.4), London 22.08 years (SEM 1.1), St Louis 18.17 years (SEM 0.8). These mean age differences were statistically significant (ANOVA: F = 9.82 df 7, 841 p < 0.001. Tukey Post hoc test: St Louis, London, Dublin, Aarhus, < London, Dublin, Aarhus Cardiff, Birmingham, Lausanne < Cardiff, Birmingham, Lausanne, Bonn).

However, there were no significant differences between sites for the numbers of men and women recruited (see Table 1) (chi squared test = 6.83 df 7 p = non significant (ns)).

### Number of probands, siblings and other relatives recruited by site

Although study participants were mainly affected proband/sibling pairs, there were a few families where parents were also included. The numbers of probands, siblings and parents recruited per site is shown in Table 2.

**Table 2:** Number of probands and siblings recruited from each site

Site	Gender	Number of probands	Number of siblings	Number of parents
Aarhus	Female	11	17	2
	Male	12	6	0
Birmingham	Female	42	53	9
	Male	18	21	3
Bonn	Female	38	43	5
	Male	12	12	0
Cardiff	Female	41	42	2
	Male	17	23	1
Dublin	Female	54	51	5
	Male	14	28	2
Lausanne	Female	18	25	0
	Male	10	3	0
London	Female	37	41	2
	Male	13	18	0
St Louis	Female	54	63	15
	Male	21	33	7

In total there were 369 families with 2 affected siblings, 36 families with 3 affected siblings, 7 families with 4 affected siblings, and 2 families with 5 affected siblings. In addition 53 parents were also interviewed and provided blood for DNA extraction. Thus there were 470 affected sibling pairs (calculated as number of pairs per family equals number of affected siblings minus 1).

### Gender, age at interview, age at illness onset and marital status for all sites combined

Of the 944 subjects, 670 (71%) were female and 274 (29%) were males and hence, the female/male ratio was 2.45:1.

Mean age at interview for all female subjects was 45.40 years (SEM 0.5) and for all males subjects was 45.69 (SEM 0.8). There were no significant gender differences for age at interview (t = -0.33, df = 477.58, p = ns)

The mean age of illness onset for depressed male subjects was 22.61 years (SEM 0.7) compared to 22.52 years (SEM 0.4) for depressed female subjects. There was no significant sex difference for age of onset.(t = -0.11, df = 443.55 p = ns).

Fifty five percent of male subjects and 61 % female subjects were living with a partner (married or cohabiting), while 45 % male subjects and 39% female subjects were living alone (ie separated, widowed, divorced or never married). Female subjects were significantly more likely to be living with a partner compared to male subjects. (Chi squared test = 26.89 df = 1 p < 0.001).

### Gender, age at interview, age of illness onset and marital status for probands, siblings and parents

There were 295 female and 117 male probands, 335 female and 144 male siblings and 40 female and 13 male parents included in the total sample. There were no significantly differences for the gender of probands, siblings or parents (chi squared test = 0.85, df = 2 p = ns).

The mean age at interview for probands was 45.94 years (SEM 0.6) and for siblings was 45.80 years (SEM 0.5). There were no significant differences for age at interview between probands and their siblings (t = 0.17, df = 872.95 p = ns)

Probands gave a mean age of illness onset of 20.22 years (SEM 0.6) while siblings reported a mean age of onset of 21.04 years (SEM 0.6). Again these differences were not statistically significant (t = -0.98, df = 882.93, p = ns)

There were also no significant differences between probands and their siblings for marital status; 161 probands and 170 siblings were living alone while 242 probands and 290 sibings were living with a partner (chi squared test = 0.81 df = 1 p = ns).

### Genotyping checking

Genotyping was carried out by DeCode and the results checked for mis-specified relationships by the programs RELPAIR and Graphical Representation of Relationships (GRR) at the Institute of Psychiatry. RELPAIR compares the multipoint probability of the data conditional on the possible relationships, while GRR calculates the IBS mean and SD for each pair and plots these values, representing each type of relative pair using a different colour. Decisions about each problem pair were made on the basis of the results from both programs, although where there was discrepancy between the programs the GRR results were used.

To check genotypes with Mendelian and other pedigree errors the PEDSTAT and MERLIN programs were used.

These data cleaning processes resulted in 929 individuals being genotyped at 932 autosomal markers and 44 X chromosome markers. Success rates for the autosomal markers were above 61% and for 90% were above 86%. For the X chromosome the success rate was above 66%. For individuals the genotyping success rate was above 73% for autosomal markers and 61% for the X chromosome.

## Discussion

### Inter-site differences and similarities

The Depression Network study has recruited affected sibling pairs and some of their parents from 7 European and 1 North American site for a linkage analysis of recurrent unipolar depression. Because of differences in local service organisation, different recruitment strategies have been employed at the different sites. This may account for the significant differences for age at interview and age at illness onset between sites. The Bonn site recruited the oldest sibling pairs, both in terms of when subjects were interviewed and also when their illnesses had commenced. The Bonn subjects had a mean age at interview of 51.46 years compared to the Dublin subjects whose mean age at interview of 43.31 years was the youngest. Similarly the Bonn subjects mean age at illness onset was 27.28 years compared to a mean age of illness onset nearly a decade earlier for the St Louis subjects (18.17 years). It is noteworthy however that the St Louis sample included several large affected sibships. Subjects from families where there are many affected relatives may have a more genetic form of the disorder that might be contributing to an earlier age of onset.

Despite these inter-site differences, the results show that there are also considerable similarities across the sites for the subjects recruited. Subjects have been mainly interviewed in their mid 40s and have experienced the onset of their recurrent depression in their early to mid 20s. Consequently subjects had on average around 20 years of history of episodes of depression when interviewed.

### Gender ratio and similarlities between probands and siblings

As expected the study has shown the same preponderance of female to male subjects as many other studies with a gender ratio of around 2.45:1 [4]. However compared to male subjects, female subjects were significantly more likely to be living with a partner rather than alone.

We would also not expect to find any significant differences between probands and siblings in terms of gender, age at interview, age at illness onset or marital status, which the results show is the case. Indeed for the purpose of finding genes for depression we would require siblings to have experienced similar forms of the illness.

### Genotyping checking

Although some subjects were excluded following genotyping due to errors that could not be reconciled, this preliminary genome linkage screen was able to include 929 subjects (98.4%) genotyped at 932 autosomal and 544 X chromosome markers. The results of the whole genome screen will be presented in due course.

## Conclusions

The Depression network study is the first co-ordinated international collaboration of its kind on the genetics of depression and one of the largest ever neuropsychiatric linkage study collection to use a uniform methodology to define and describe the phenotype. Despite taking place across eight sites and in six different countries good inter-rater agreement has been achievable as has good comparability of data.

The study has been designed to overcome the difficulties that have been encountered in linkage studies of other psychiatric disorders such as schizophrenia and bipolar disorder. These started out optimistically with the assumption that genes of large effect would exist in at least some multiply affected families. However, after over a decade of contradictory findings and non replications, there is now consensus that such families are very rare or perhaps nonexistent. Rather it seems likely that common familial psychiatric disorders result from the combined effect of multiple genes none of which is either necessary or sufficient to cause the condition [20]. Consequently large samples are required to have adequate power to detect genes of comparatively small effect, typically where the "risk genotype" confers a genotype relative risk of less than two. Some order is beginning to emerge as a result of meta-analyses of schizophrenia and bipolar data [21-23] however meta-analyses are fraught with difficulties resulting from differences in diagnostic methods, types of the family, genetic marker sets used and methods of ascertainment, in addition to the technical problems of how best to assess statistical significance. It is far preferable to have large diagnostically and ethnically homogenous data sets such as the one described here which will ultimately contain well over 1000 families. Family samples of a comparable size are also being collected elsewhere [11]. Until now studies of the genetics of unipolar depression have lagged behind those on schizophrenia and bipolar disorder but in doing so we have been able to learn from earlier mistakes. With hope therefore, uncovering the molecular genetic basis of unipolar depression promises to throw up less uncertainties and produce more consistency than has been characteristic of linkage and association studies in psychiatry.

## Competing interests

This study was funded by 3 year research grants to each participating site from Glaxo Wellcome Research and Development.

## Authors' contributions

AF & PMcG were overall study Principal Investigators (PIs), conceived the study and were co-ordinators of the study design, diagnostic reliability and data analysis. AF trained the interviewers from all the sites and wrote the paper. SB, LM and JP obtained the funding from Glaxo Wellcome, recruited site PIs and oversaw the quality of data collection, handling and analysis. GB analysed the genotyping data. The following authors were the individual site PIs in charge of all aspects of subject recruitment and data quality locally: OM Aarhus site, NC, LJ and IJ (joint) Birmingham site, MR and WM (joint) Bonn site, AK and MO (joint) Cardiff site, MG Dublin site, MP Lausanne site AF and PMcG (joint) London site and TR St Louis site. All authors have read and approved the contents of the final manuscript.

## Pre-publication history

The pre-publication history for this paper can be accessed here:


